# Willingness to Receive a COVID-19 Vaccine and Associated Factors among Older Adults: A Cross-Sectional Survey in Shanghai, China

**DOI:** 10.3390/vaccines10050654

**Published:** 2022-04-21

**Authors:** Linlin Wu, Xiaolan Wang, Ruiping Li, Zhuoying Huang, Xiang Guo, Jiechen Liu, Han Yan, Xiaodong Sun

**Affiliations:** 1 Department of Immunization Program, Shanghai Municipal Center for Disease Prevention and Control, Shanghai 200336, China; wulinlin@scdc.sh.cn (L.W.); huangzhuoying@scdc.sh.cn (Z.H.); guoxiang@scdc.sh.cn (X.G.); liujiechen@scdc.sh.cn (J.L.); yanhan@scdc.sh.cn (H.Y.); 2 Department of Epidemiology, School of Public Health, Fudan University, Shanghai 200032, China; 21211020019@m.fudan.edu.cn; 3 Department of Immunization Program, Fengxian District Municipal Center for Disease Prevention and Control, Shanghai 201499, China; liruiping112@163.com

**Keywords:** COVID-19, willingness, older adults, vaccine, China

## Abstract

Background: Older individuals have a high risk of morbidity and mortality due to COVID-19, and one of the most effective ways to prevent COVID-19 is vaccination. Little is known about older people’s willingness to receive a COVID-19 vaccine. Therefore, the objective of this study was to assess the acceptance of and factors influencing the intention to receive a COVID-19 vaccination among older adults in Shanghai, China. Methods: A cross-sectional study was conducted among older adults (≥60 years old) in Shanghai. Face-to-face interviews with a questionnaire were conducted in community health service centers, recording several parameters: demographic information, health-related factors; perceived likelihood, severity, and burden of COVID-19; perceived safety, effectiveness, necessity, and benefit of the COVID-19 vaccine, as well as their trust in the vaccine delivery system and doctors; willingness to receive a COVID-19 vaccination. Bivariate analysis between several survey items and the willingness to receive a COVID-19 vaccination was conducted using a chi-square test. Logistic regression was used to assess to what degree each variable affected the willingness to receive a COVID-19 vaccination. Results: Of the 1067 participants, 90.91% (970/1067) confirmed that they were willing to receive a COVID-19 vaccination. The participants were more likely to be willing to be vaccinated if they were immigrants (OR = 1.988, 95%CI = 1.062–3.717), had an education level of junior high school (OR = 2.724, 95%CI = 1.000–7.423) or high school or above (OR = 3.993, 95%CI = 1.576–10.119), and had a monthly income of CNY 3000–5000 (OR = 32.770, 95%CI = 1.144–6.711) or CNY >5000 (OR = 2.309, 95%CI = 1.003–5.319). The participants were also more likely to be willing to be vaccinated if they had received a pneumonia vaccination previously (OR = 2.138, 95%CI = 1.016–4.500), perceived the safety of the COVID-19 vaccine (OR = 1.508, 95%CI = 1.073–2.119), perceived the necessity of the COVID-19 vaccine (OR = 2.604, 95%CI = 1.946–3.484), or trusted the suggestions of doctors (OR = 1.706, 95%CI = 1.115–2.618). The participants were less likely to be willing to be vaccinated if they were aged ≥76 years (OR = 0.498, 95%CI = 0.264–0.939), reported a physical health condition of bad or below (OR = 0.229, 95% CI = 0.095–0.549), or were worried about the adverse effects of a COVID-19 vaccination (OR = 0.503, 95%CI = 0.364–0.695). Conclusions: Under the free vaccination policy for COVID-19, older adults have a high intention to be vaccinated to prevent COVID-19 in Shanghai, China. Widely publicizing the safety and necessity of COVID-19 vaccination is necessary.

## 1. Introduction

Coronavirus disease 2019 (COVID-19) is an infectious disease caused by the severe acute respiratory syndrome coronavirus 2 (SARS-CoV-2) [1,2]. As of 20 February 2022, over 422 million confirmed cases and over 5.8 million deaths have been reported globally [3]. The population is commonly susceptible to SARS-CoV-2, especially the elderly with comorbidities. Older adults and those with underlying diseases such as cardiovascular disease, diabetes, chronic respiratory diseases, or cancer who are infected with SARS-CoV-2 are more likely to develop serious illness. The case fatality rates among those aged <50 years are typically <0.5%, compared to 1.3% in people 50–59 years old, 3.6% in 60–69 years old, 8.0% in 70–79 years old, and 14.8% in people ≥80 years old [4]. Age and co-morbid medical conditions have also been reported as important risk factors for severe COVID-19 [5,6].

COVID-19 vaccines are highly effective at preventing severe illness, hospitalizations, and death. Studies have shown that vaccines have played an important role in preventing COVID-19 hospitalizations and deaths among adults over 60 years old [7,8,9,10]. However, a lower vaccine acceptance rate would be insufficient to prevent disease transmission. Studies have found that people have various concerns regarding the COVID-19 vaccine, and the rate of willingness to accept COVID-19 vaccination has decreased as the pandemic progressed. Some factors have contributed to the hesitancy, such as socioeconomic characteristics (age, gender, etc.), perception of the safety and efficacy of vaccines, perception of risks and benefits associated with the COVID-19 vaccination, etc. [11,12,13,14,15].

Shanghai is China’s major eastern gate to the outside world and is the first city to enter the aging population stage in China. At the end of 2020, the number of people aged 60 years and above in Shanghai’s population reached 5.18 million, accounting for 35.2% of the total population, and the life expectancy of the city residents was 83.63 years old. These findings highlight the need for health-related strategies to cope with an aging society [16].

China provides free COVID-19 vaccinations for the entire population, step by step [17]. In March and May 2021, Shanghai launched a COVID-19 vaccination program for people aged 60–75 years old and over 76 years old. Vaccination for the elderly has been the focus of epidemic prevention and control. People’s vaccination willingness determines whether or not they receive a COVID-19 vaccination. Due to their health status or other socioeconomic factors, the elderly may have a different acceptance of the COVID-19 vaccination compared with other age group adults. Studies in other countries such as the US found higher acceptability of the elderly, but hesitancy for COVID-19 vaccination is a relevant problem in older people, particularly in those with a low level of income and education [14,18,19,20,21]. A survey conducted in Saudi Arabia showed that the vaccination acceptance rate among elderly adults is low [22]. Currently, although studies related to the willingness of older adults to receive vaccination have been conducted in other countries around the world, there is a lack of relevant studies on older adults in China. Therefore, this study aimed to assess the acceptance of the need for, the attitude toward, and the determinants of COVID-19 vaccination among older adults in Shanghai, China.

## 2. Materials and Methods

### 2.1. Setting and Study Design

A cross-sectional survey was conducted in 4 of the 16 districts in Shanghai. These four districts were randomly selected, including two rural districts and two urban districts. Subjects were enrolled by a convenience sampling among those visiting community health service centers for physical examination. People were invited to voluntarily participate in the survey by responding to a face-to-face interviews, which were conducted by trained field workers at community health service centers from May to June 2021. The eligibility criteria were being aged 60 years or above, being fluent in Mandarin, and currently living in Shanghai.

### 2.2. Sampling

With a land surface area of 6340 km^2^ and around 581.5 million elderly inhabitants aged ≥60, Shanghai lies in the eastern of China. The study size was calculated using the sample size calculation formula, *n* = Zα^2^ pq/d^2^. The parameters used for the calculation were set as follows: A ratio of vaccine acceptance in the older group (p) at 0.77, which was the rate obtained from other studies performed in China. Therefore, with q = 1 − p, q was 0.23, with an alpha error of 0.01, and Zα = 2.56, d; the tolerance error was 0.05, and *n* was calculated to be 465. A 0% dropout rate was added, which resulted in *N* of 510 for females and males. We collected data on 1100 adults over the age of 60 years, which yielded a sample of 1067 respondents after deleting cases with missing data, with a response rate of 97.0%.

### 2.3. Questionnaire

A self-administrated questionnaire was developed based on past research involving vaccination behaviors [23,24,25,26]. It was tested during a pilot study and proven by experts to be reliable and valid. The time spent on the completion of the questionnaire was approximately 10–15 min. To ensure the items of the questionnaire clarify, the questionnaire was pilot-tested with 30 elderly adults, and minor revisions were made. The pilot-test surveys were not included in the analysis. The reliability index was assessed for both the pilot and original study by using Cronbach’s alpha (internal consistency coefficient). The alpha values were 0.83 and 0.81, respectively, showing a satisfactory level of reliability [27].

The questionnaire included five major items: (1) demographic information, such as gender, age, education level, immigration status, marital status, and monthly income; (2) health-related factors, including health insurance, history of influenza vaccination, history of pneumonia vaccination, previous adverse reactions, and self-reported physical health condition, used a 3-point scale; (3) perceived likelihood, severity, and burden of COVID-19. A 5-point Likert scale (e.g., “very high” to “very low” on infection risk, severity, and burden) was used to assess each item; (4) perceived safety, effectiveness, necessity, and benefit of the COVID-19 vaccine, as well as their trust in the vaccine delivery system and doctors. A 5-point response scale ranging from “very” to “not at all” was also used to access, and (5) willingness to receive a COVID-19 vaccination. We assessed the participants’ vaccination willingness by asking whether they were willing to receive a COVID-19 vaccination or not; participants who wanted to get the COVID-19 vaccine were considered as willing to accept.

### 2.4. Statistical Analysis

SPSS 17.0 (SPSS Inc., Chicago, IL, USA) was used for the data analyses. Descriptive statistics were performed to describe the demographic characteristics and health-related factors of participants. Categorical data are described using frequencies and percentages. For describing the continuous data, means and standard deviations (SDs) are used. A Pearson’s chi-square test was used to detect the associations between willingness and the demographic and health-related variables, and Cramer V was calculated. We also used regression models with robust standard errors to identify correlates of COVID-19 vaccine acceptability [28]. We entered variables with significance in bivariate analyses into an initial multivariable model and then used a backward selection procedure to create a final multivariable model, which was performed to access the associations between each independent variable and willingness to receive a COVID-19 vaccination, with odds ratios (ORs) and 95% confidence intervals (CIs) being calculated. All reported *p*-values are based on two-tailed tests and were considered statistically significant at the level of 0.05 or less.

### 2.5. Ethical Considerations

This study was approved by the Ethical Review Board of the Shanghai Municipal Center for Disease Control and Prevention on 2 May 2020 (2020-32).

## 3. Results

### 3.1. Participants’ Characteristics

Among 1067 respondents, most participants were between 60 and 75 years old (75.07%), and the mean age of the participants was 71.3 ± 5.6 (51.27%). Just over half of the participants were female (51.27) and part of the resident population (50.33%). Regarding educational background, only 30.27% (*n* = 323) had an associate degree or above. In terms of monthly income, 40.39% (*n* = 431) of the respondents had a monthly income ranging from CNY 5000 to 8000. Most respondents had some type of health insurance (94.94%), and 60.64% reported that their physical health conditions were good or very good. In terms of vaccination history, only 14.81% of the respondents had received vaccinations against influenza in the past three years, and 43.86% reported that they had received a pneumonia vaccination previously (Table 1).

### 3.2. Acceptance of the COVID-19 Vaccine

Overall, 90.9% (*n* = 970) of the study participants indicated acceptance of the need for a COVID-19 vaccination, while 9.1% (*n* = 97) expressed unwillingness to receive it.

Among those who would accept vaccination, 67.22% (*n* = 652) were more likely to be vaccinated with domestic vaccines, while only 3.50% (*n* = 34) were willing to be vaccinated with imported vaccines, and 29.28% (*n* = 284) were willing to vaccinate with either domestic or imported vaccines.

Among the 97 subjects who were unwilling to be vaccinated, 37 (38.14%) were worried about a serious adverse reaction following vaccination, and 23 (23.71%) were worried about the safety of the vaccine. Fifteen people (15.46%) considered it unnecessary to be vaccinated.

### 3.3. Influencing Factors of Vaccination Acceptance

A comparison of the baseline characteristics between the two groups with a chi-square test is displayed in Table 2. Immigration status (immigrants), education (junior high school, high school, or above), monthly income (CNY > 5000 and 3000–5000), self-reported physical health condition (good or above), and having received influenza and pneumonia vaccinations were associated with higher acceptance of the COVID-19 vaccine (Table 2).

In addition, our survey covered a few key variables that are relevant to the awareness of COVID-19 and the need for a COVID-19 vaccine (Table 3). We found that respondents who perceived higher safety of, higher effectiveness of, higher necessity for, and higher benefit of the COVID-19 vaccine were significantly more likely to indicate vaccine acceptance. Those participants who ascribed higher importance to vaccination convenience and demonstrated a stronger reaction toward vaccination doses were less likely to express acceptance. Trust in their doctor’s suggestion was positively associated with vaccine acceptance.

The multivariable logistic regression analysis revealed that factors predicting willingness to vaccinate against COVID-19 were being an immigrant (OR = 1.988, 95%CI = 1.062–3.717), having an education level of junior high school (OR = 2.724, 95%CI = 1.000–7.423) and high school or above (OR = 3.993, 95%CI = 1.576–10.119), having a monthly income of CNY 3000–5000 (OR = 32.770, 95%CI = 1.144–6.711) or CNY >5000 (OR = 2.309, 95%CI = 1.003–5.319), having received a pneumonia vaccination previously (OR = 2.138, 95%CI = 1.016–4.500), perceiving the safety of the COVID-19 vaccine (OR = 1.508, 95%CI = 1.073–2.119), perceiving the necessity for a COVID-19 vaccine (OR = 2.604, 95%CI = 1.946–3.484), and trust in the suggestion of a doctor (OR = 1.706, 95%CI = 1.115–2.618). Factors predicting a lack of willingness to vaccinate were being ≥75 years old (OR = 0.498, 95%CI = 0.264–0.939), having a self-reported physical health condition of bad or below (OR = 0.229, 95%CI = 0.095–0.549), and worrying about the adverse effects of the COVID-19 vaccination (OR = 0.503, 95%CI = 0.364–0.695). The likelihood ratio and R^2^ for the final model were 21.799 and 0.411, respectively (Table 4).

## 4. Discussion

The COVID-19 pandemic has caused unprecedented losses for the world [29,30]. The COVID-19 vaccination is considered to be a way to prevent and control the spread of COVID-19. China is still faced with the risk of continuous import through multiple channels and local outbreaks [31]. The elderly and people with chronic diseases are at high risk of severe illness and death after infection with COVID-19. In China, a COVID-19 vaccine has been provided and accepted on a large scale among adults aged 18–59 years old, and it is crucial to include the elderly and children in the vaccination program [32]. Understanding the willingness of the elderly aged ≥60 years to receive a COVID-19 vaccination and timely intervention targeting the factors that affect the willingness to be vaccinated and actually being vaccinated will play an important role in further improving the vaccination rate of those ≥60 years old. One META analysis published in December 2021 addressing vaccine hesitancy among older adults searched the relevant literature up to June 2021 and included 15 articles; five were from Asian countries, excluding China [18]. Only one article published in December 2021 exploring vaccination intentions of Chinese older adults aged 60 years or older was found after our literature search [33]. Therefore, there are areas of research on vaccination intentions of older adults that need to be further explored, especially in China [34]. The aim of this study was to explore the willingness to receive vaccinations and identify the factors associated with the intention of older adults to be vaccinated against COVID-19, the results from which will help guide the strategy for vaccination and promote a positive attitude toward vaccination for the elderly in China. The results will also provide a scientific basis for improving the willingness of older adults to receive vaccinations in other countries around the world to some extent.

At the time of evaluation, approximately 91% of the surveyed participants were willing to receive a COVID-19 vaccination, with only 9% of this population reporting no willingness to be vaccinated in the future. Our findings are in line with the vaccine acceptance found in the US and in southern Italy [35,36]. Compared to the general population, older adults may have a higher vaccination intention [37,38], as the COVID-19 vaccination rate among the general population aged 18 years and older ranged from 60.4% to 83.8% [34,39,40,41]. We also found that among the elderly population willing to be vaccinated, more people preferred to use domestic vaccines, coinciding with a previous study that suggested that 64.2% of the surveyed population preferred to use domestic vaccines [42,43]. The higher willingness to vaccinate among the elderly in this study may be due to the following reasons: first, the clinical trials for the vaccine are more complete at this stage, and its safety and reliability are guaranteed; second, the elderly population may be more willing to receive a vaccination due to their susceptibility and the threat of comorbidities. Previous studies have also shown that being married is associated with a higher willingness to receive a vaccination [44], and the fact that 70% of the elderly population surveyed in this study were married may also have had some influence.

We also identified several independent factors associated with acceptance, including age, immigration status, educational level, monthly income, self-reported physical health condition, history of pneumonia vaccination, perceived safety of the COVID-19 vaccine, perceived necessity for the COVID-19 vaccine, worry about the adverse effects of COVID-19 vaccination, and trust in the suggestion of a doctor.

We found that being an immigrant was a positive factor in the intention to receive a COVID-19 vaccination, which is consistent with another study [45]; we assume that the low intention to be vaccinated among the resident population might be due to the mistrust caused by some negative reports on vaccine safety and the greater attention toward vaccine safety. Previous research has found that lower economic levels may be associated with higher vaccination intentions [37], a finding that is at odds with our survey. People with a higher education level may have a more comprehensive understanding of the benefits of vaccines and, thus, be able to objectively and rationally perceive the protective effects of vaccines and be more willing to receive them, which is also consistent with the results of previous studies [22,44,46]. Some studies [47,48] have reported that the safety and effectiveness of vaccines are important factors in determining COVID-19 vaccination rates. This study also showed that the perceived safety and the necessity for a COVID-19 vaccine were significantly influencing factors, whereas the perceived susceptibility to and severity of infection were not associated with vaccine acceptance. The COVID-19 vaccine acceptance increased when the older population’s awareness of vaccine safety increased, which is consistent with other studies [49] that reported that perception of the potential harm of a COVID-19 vaccine decreased the acceptance of receiving a vaccination. Thus, improving older adults’ awareness of the COVID-19 vaccine safety is conducive to increasing the willingness to receive a COVID-19 vaccination.

In our survey, 80.88% of the respondents reported that they had chronic medical conditions, and 39.36% assessed their physical health condition as moderate or bad. The willingness rate of respondents reporting their physical health condition as good was higher than those reporting it as moderate or bad. Those who self-reported poor physical condition were worried that vaccination would cause serious adverse effects. The Technical Guidelines for New Coronavirus Vaccination (First Edition) [50] recommended that people aged ≥60 years with chronic diseases receive a COVID-19 vaccination under stable drug control and good health conditions. Therefore, these elderly people should inform the vaccination personnel in detail about their health conditions; after a comprehensive evaluation, it can be decided whether to vaccinate or to postpone the vaccination. At the same time, vaccination personnel need to strengthen their relevant knowledge and strictly grasp the contraindications of vaccination for such elderly people.

The results also showed that some elderly people were still reluctant to receive a COVID-19 vaccination, mainly because they were worried about the safety of the vaccine and the potential for a serious adverse reaction after vaccination. This suggested that we should emphasize the necessity for and safety of the COVID-19 vaccine among the elderly. At the same time, a professional should actively recommend and guide the elderly to vaccination.

The novelty of this study was its investigation of the willingness of elderly people to be vaccinated and the possible associated influencing factors. However, this study has some limitations. First, participants were enrolled by convenience sampling in four districts of Shanghai, and the final sample is not representative of the whole population of the Shanghai elderly. Secondly, the willingness to vaccinate was mainly self-reported, which was more subjective. A more objective assessment indicator could be considered subsequently to explore the vaccination willingness and its influencing factors in a nationally representative group of older adults. Thirdly, the acceptability might vary with different types of COVID-19 vaccines, and our study did not differentiate between the different types of COVID-19 vaccines [43]. The willingness to vaccinate against different types of COVID-19 vaccines is worth further exploration. Additionally, there is also a study conducted in Italy that suggests that green code access policies may have an effect on vaccination intentions [36]; our study did not take green code access policies into account. Future studies and investigations are needed to monitor the public perception, acceptability, and uptake of COVID-19 with the progress of vaccination programs and access to the effects of vaccination programs in terms of different policies.

## 5. Conclusions

This study found that the elderly population in Shanghai, China, had high intentions to be administered a COVID-19 vaccination. In addition, immigrant populations and those with a history of vaccination may have higher intentions. Focusing on low-education, low-income populations and conducting physician-led campaigns on the safety of and necessity for the vaccine could help increase the willingness of older adults to receive it.

## Figures and Tables

**Table 1:** Demographic and health-related characteristics of participants.

Variables	Total Sample (*n* = 1067)
Demographic characteristics	
Age	
60–75 years old	801 (75.07)
≥76 years old	266 (24.93)
Gender	
Males	520 (48.73)
Females	547 (51.27)
Immigration status	
Residents	537 (50.33)
Immigrants	530 (49.67)
Education	
Middle school and below	441 (41.33)
High school	303 (28.40)
Associate, Bachelor’s, or above	323 (30.27)
Marital status	
Married	808 (75.73)
Single, divorced, or widowed	259 (24.28)
Monthly income	
<3000	414 (38.80)
3000–5000	431 (40.39)
≥5000	222 (20.80)
Health-related characteristics	
Health insurance	
Have	1013 (94.94)
Do not have	54 (5.06)
History of influenza vaccination	
Yes	158 (14.81)
No	909 (85.19)
History of pneumonia vaccination	
Yes	468 (43.86)
No	599 (56.14)
Previous adverse reaction	
Yes	77 (7.22)
No	990 (92.78)
Self-reported physical health condition (current)	
Good or above	647 (60.64)
Moderate	352 (32.99)
Bad or below	68 (6.37)
Self-reported chronic medical conditions ^1^	
Yes	863 (80.88)
No	204 (19.12)

^1^ Chronic medical conditions, such as diabetes, asthma, or heart disease.

**Table 2:** Differences in willingness to receive a COVID-19 vaccination between different demographic characteristic factors.

Variables	Willing to Receive a COVID-19 Vaccination (*n* = 970)	Unwilling to Receive a COVID-19 Vaccination (*n* = 97)	*χ* ^2^	*p*	OR (95%CI)	Cramer V
Demographic characteristics						
Age			3.704	0.054		0.059
60–75 years old	736 (75.88)	65 (67.01)			Ref.	
≥75 years old	234 (24.12)	32 (32.09)			1.548 (0.989–2.424)	
Gender			3.106	0.078		−0.054
Males	481 (49.59)	39 (40.21)			Ref.	
Females	489 (50.41)	58 (59.79)			1.462 (0.956–2.237)	
Immigration status			14.996	<0.001		−0.119
Residents	470 (48.45)	67 (69.07)			Ref.	
Immigrants	500 (51.55)	30 (30.93)			0.421 (0.269–0.659)	
Education			42.195	<0.001		
Less than junior high school degree	373 (38.45)	68 (70.10)			Ref.	0.192
Junior high school	283 (29.18)	20 (20.62)			2.580 (1.531–4.347)	
High school or above	314 (32.37)	9 (9.28)			6.360 (3.123–12.953)	
Marital status			1.237	0.266	0.770 (0.485–1.224)	−0.034
Married	739 (76.20)	69 (71.13)				
Single, divorced, or widowed	231 (23.80)	28 (28.87)				
Monthly income			22.650	<0.001		
<3000	355 (36.60)	59 (60.82)			Ref.	0.146
3000–5000	409 (42.16)	22 (22.68)			3.090 (1.856–5.144)	
≥5000	206 (21.24)	16 (16.50)			2.140 (1.200–3.816)	
Health-related characteristics						
Health insurance			0.002	0.965		0.001
Have	921 (94.95)	92 (94.85)			Ref.	
Do not have	49 (5.05)	5 (5.15)			1.021 (0.397–2.628)	
History of influenza vaccination			7.882	0.005		0.086
Yes	153 (15.77)	5 (5.15)			Ref.	
No	817 (84.23)	92 (94.85)			3.446 (1.378–8.616)	
History of pneumonia vaccination			27.747	<0.001		0.161
Yes	450 (46.39)	18 (18.56)			Ref.	
No	520 (53.61)	79 (81.44)			3.798 (2.242–6.435)	
Previous adverse reaction			1.524	0.217		0.038
Yes	73 (7.53)	4 (4.12)			Ref.	
No	897 (92.47)	93 (95.88)			1.892 (0.676–5.294)	
Self-reported physical health condition (current)			59.354	<0.001		
Good or above	616 (63.50)	31 (32.00)			Ref.	0.236
Moderate	307 (31.65)	45 (46.40)			0.343 (0.213,0.554)	
Bad or below	47 (4.85)	21 (21.60)			0.113 (0.060,0.211)	
Self-reported chronic medical conditions			0.155	0.694		0.012
Yes	786 (81.03)	77 (79.38)			Ref.	
No	184 (18.97)	20 (20.62)			1.110 (0.661,1.862)	

Note: COVID-19—coronavirus disease 2019; OR—odds ratio; CI—confidence interval.

**Table 3:** Factors associated with attitude scores toward COVID-19 disease and COVID-19 vaccination among older people in Shanghai.

	Willing Mean (SD)	Unwilling Mean (SD)	*p*	Bivariate OR (95%CI)
Perceived likelihood of COVID-19 infection	2.15 (1.00)	2.23 (1.14)	0.432	0.923 (0.756–1.127)
Perceived severity of COVID-19 infection	3.08 (1.14)	3.13 (1.25)	0.648	0.959 (0.799–1.149)
Perceived burden of COVID-19 infection	3.75 (0.98)	3.65 (1.08)	0.356	1.104 (0.895–1.362)
Perceived safety of the COVID-19 vaccine	3.71 (0.91)	3.11 (0.97)	<0.001	1.969 (1.575–2.463)
Perceived effectiveness of the COVID-19 vaccine	4.14 (0.80)	3.49 (0.96)	<0.001	2.494 (1.923–3.236)
Perceived necessity for a COVID-19 vaccine	3.89 (1.01)	2.77 (0.97)	<0.001	2.146 (1.818–2.532)
Perceived benefit of the COVID-19 vaccine	4.30 (0.85)	3.41 (0.86)	<0.001	2.967 (2.304–3.817)
Worry about adverse effects of COVID-19 vaccination	3.07 (1.06)	2.52 (0.87)	<0.001	0.612 (0.501–0.748)
Trust in the vaccine delivery system	3.68 (0.89)	3.55 (0.87)	0.167	1.185 (0.932–1.508)
Trust in the suggestion of a doctor	4.17 (0.95)	3.42 (0.85)	<0.001	2.083 (1.692–2.571)
Importance of vaccination convenience	2.31 (1.02)	2.77 (1.01)	<0.001	0.654 (0.536–0.797)
Impact of vaccination doses on willingness	2.29 (1.00)	2.69 (0.97)	<0.001	0.674 (0.549–0.828)

Note: COVID-19—coronavirus disease 2019; OR—odds ratio; SD—standard deviation; CI—confidence interval.

**Table 4:** COVID-19 vaccination acceptance of older people by multivariate logistic regression analysis.

	OR (95%CI)	*p*
Immigration status		
Residents	Ref.	
Immigrants	1.988 (1.062–3.717)	0.032
Age		
60–75 years old	Ref.	
≥75 years old	0.498 (0.264–0.939)	0.031
Education		
Less than junior high school degree	Ref.	
Junior high school	2.724 (1.000–7.423)	0.050
High school or above	3.993 (1.576–10.119)	0.004
Monthly income		
<3000	Ref.	
3000–5000	2.770 (1.144–6.711)	0.024
>5000	2.309 (1.003–5.319)	0.049
Self-reported physical health condition (current)	Ref.	
Good or above		
Moderate	0.444 (0.192–1.028)	0.058
Bad or below	0.229 (0.095–0.549)	0.001
History of influenza vaccination		
No	Ref.	
Yes	2.414 (0.757–7.698)	0.137
History of pneumonia vaccination		
No	Ref.	
Yes	2.138 (1.016–4.500)	0.045
Perceived safety of the COVID-19 vaccine	1.508 (1.073–2.119)	0.018
Perceived effectiveness of the COVID-19 vaccine	1.595 (0.961–2.646)	0.071
Perceived necessity for a COVID-19 vaccine	2.604 (1.946–3.484)	<0.001
Perceived benefit of the COVID-19 vaccine	1.511 (0.878–2.604)	0.136
Worried about adverse effects of COVID-19 vaccination	0.503 (0.364–0.695)	<0.001
Trust in the suggestion of a doctor	1.706 (1.115–2.618)	0.014
Importance of vaccination convenience	0.993 (0.580–1.701)	0.980
Impact of vaccination doses on willingness	0.853 (0.496–1.466)	0.565

Note: COVID-19—coronavirus disease 2019; OR—odds ratio relative risk; CI—confidence interval.

## Data Availability

The data presented in this study are available on request from the corresponding author. The data are not publicly available due to their containing information that could compromise the privacy of research participants.
